# Early Pregnancy Waist Circumference for Prediction of Fetal Macrosomia

**DOI:** 10.1007/s43032-025-01833-7

**Published:** 2025-03-10

**Authors:** Katja Junus, Emelie Lindberger, Heidrun Pétursdóttir Maack, Birgitta Segeblad, Inger Sundström Poromaa, Anna-Karin Wikström

**Affiliations:** Department of Women’s and Children’s Health, Uppsala University, Akademiska Sjukhuset, SE 751 85, Uppsala, Sweden

**Keywords:** Large for gestational age, Macrosomia, Prediction model, Waist circumference

## Abstract

Fetal macrosomia is associated with adverse short- and long-term outcomes for the mother and the child. Present models to predict fetal macrosomia cannot be used in all settings, and their precision could be improved. We assessed if waist circumference could replace or outperform weight for early pregnancy prediction of macrosomia. We included 5827 women in this population-based cohort study and assessed the influence of early pregnancy waist circumference and weight on the prediction of macrosomia with logistic regression analysis. We generated receiver operating characteristic (ROC) curves and calculated the area under the curve (AUC) to compare models, including waist circumference, weight, or neither of them. The odds of macrosomia increased with a larger waist circumference (adjusted odds ratio (AOR) 1.03 (95% Confidence Interval (CI) 1.02, 1.04)). For women with waist circumference between 80 and 88 cm the AOR was 1.41 (95% CI 1.09, 1.82) and women with waist circumference ≥ 88 cm had AOR 1.98 (95% CI 1.56, 2.53) for macrosomia. There was no difference in predictive capacity between waist circumference and weight in the macrosomia prediction model. The AUC was 0.75 (95% CI 0.72, 0.77) for waist circumference and 0.74 (95% CI 0.72, 0.77) for weight. The model that excluded waist circumference and weight had an AUC of 0.72 (95% CI 0.70, 0.75). The predictive capacity of the model including waist circumference was, however, higher than that of the model without waist circumference or weight (*p* < 0.001). In conclusion, waist circumference can replace weight in an early pregnancy macrosomia prediction model.

**Supplementary Information:**

The online version contains supplementary material available at 10.1007/s43032-025-01833-7.

## Background

Fetal macrosomia affects approximately 10% of pregnancies and is associated with adverse short- and long-term outcomes for the mother and the child [1]. For example, macrosomia increases the risk of cesarean section, perineal and vaginal tears, prolonged or obstructed labor, shoulder dystocia, and post-partum bleeding. For the newborn, the risks include birth asphyxia, death, hypoglycemia, neonatal resuscitation, permanent nerve injuries, and respiratory distress syndrome. Further, the child is at increased risk for obesity and type 2 diabetes mellitus later in life [2].

Given the potential adverse perinatal outcomes, there is a need to predict macrosomia. Early pregnancy macrosomia prediction could identify women in need of increased pregnancy surveillance and allow for interventions to decrease the risk. For example, structured prenatal exercise is reported to reduce the risk of having a large newborn [3]. Moreover, prediction of macrosomia could aid in planning and managing delivery.

Early pregnancy macrosomia prediction models often combine maternal history, fetal biometry, biophysical, and biochemical markers [4, 5]. The anthropometric measures included in these models are maternal height and weight. However, limited resources make such models useless in many settings. It is not only fetal biometry and biochemical markers that might be difficult to measure; there is sometimes no access to scales in low-resource settings.

Waist circumference is simple, cheap, and can be used as an alternative to maternal body mass index (BMI) or weight for assessing overweight and obesity. The measure correlates to central adiposity and visceral fat thickness and is an independent risk factor for cardiometabolic complications in non-pregnant individuals [6]. Moreover, early pregnancy waist circumference is reported to be an independent risk factor for gestational diabetes, preterm delivery, and preeclampsia [7, 8]. In addition, pre-pregnancy waist circumference is reported as a macrosomia risk factor independent of BMI among US black women [9]. Consistent with these reports, early pregnancy waist circumference has been proposed as a potentially stronger predictor for adverse perinatal outcomes than BMI or weight [10].

Here we assessed if waist circumference can replace or outperform weight in an early pregnancy macrosomia prediction model.

## Methods

### Population

We measured early pregnancy waist circumference in 5827 pregnant women at their first antenatal visit in the County of Uppsala, Sweden, between January 5, 2015 and December 29, 2017. We excluded pregnancies that ended in miscarriage, termination, or had missing birth information (*n* = 590).

Clinical data were extracted from electronic medical records and linked to the Medical Birth Register (MBR) held by the Swedish National Board of Health and Welfare using personal identity numbers. Linkage failed in 75 pregnancies, which were excluded. After linkage, the study population was pseudo-anonymized. The MBR contains prospectively collected data on all births at 22 gestational weeks or later in Sweden, including demographics and outcomes of the pregnancy. We obtained data from both the index and previous pregnancies, allowing us to identify women who had previously given birth to a newborn with macrosomia.

**Fig. 1:**
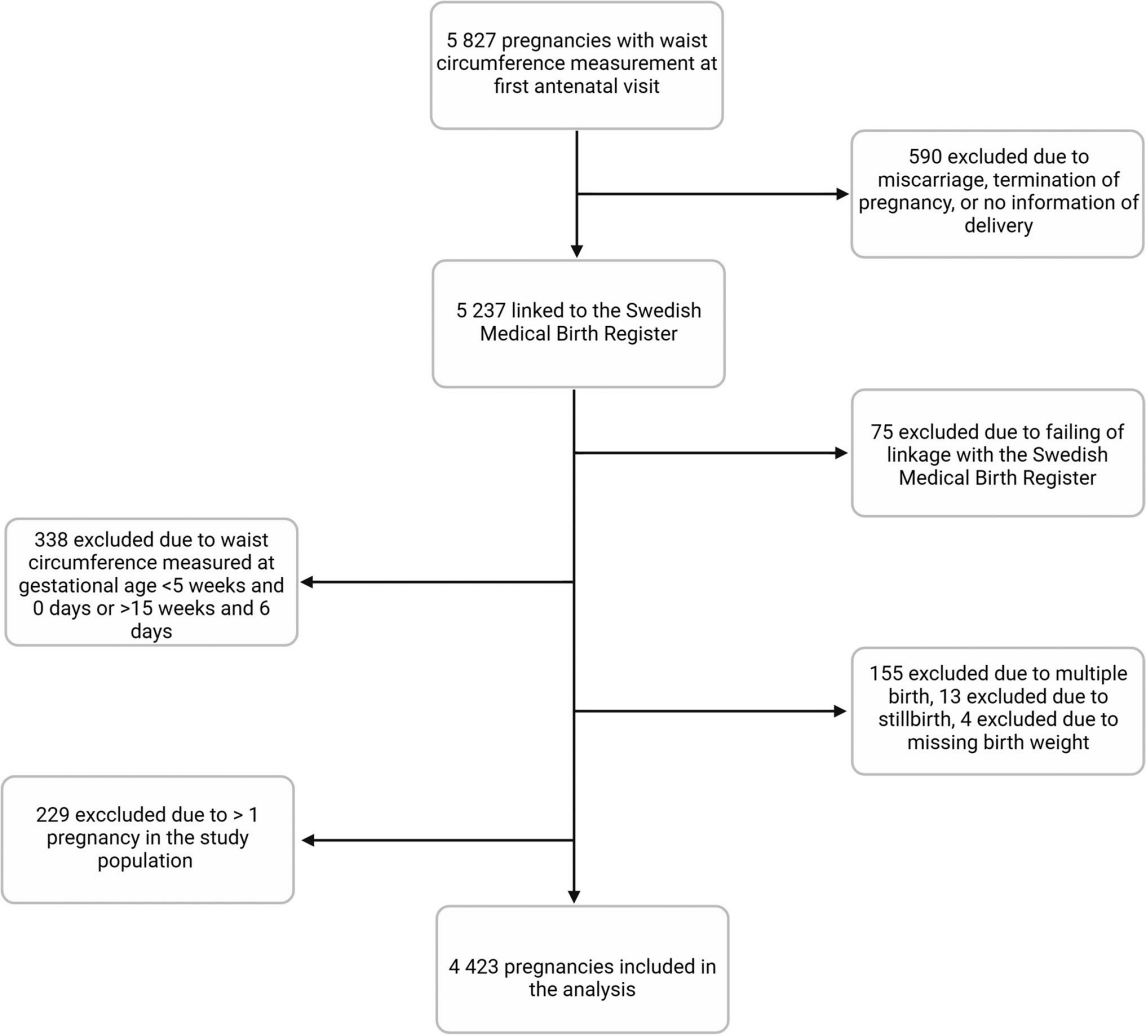
Flowchart of the study population

We excluded 338 pregnancies where waist circumference was measured at gestational ages < 5 weeks and 0 days or > 15 weeks and 6 days. Multiple pregnancies (*n* = 155), stillbirths (*n* = 13), missing birth weight (*n* = 4), and subsequent pregnancies to women already included (*n* = 229) were also excluded. Our final study population consisted of 4423 pregnancies with early pregnancy waist circumference and weight data, resulting in live births at 22 gestational weeks or later (Fig. 1).

### Exposure

Waist circumference was measured at the first antenatal visit. With the woman standing, her midwife measured the waist circumference midway between the lower rib margin and the iliac crest, using standardized measurement tapes [11]. Midwives received verbal and written information on the measurement procedure, which were repeated throughout the study period. We recorded waist circumference as a continuous variable (cm) and as a categorical variable with cutoffs ≥ 80 and ≥ 88 cm, corresponding to the WHO sex-specific cutoff points for increased (≥ 80 cm) and substantially increased (≥ 88 cm) risk of metabolic complications in the general population [11].

### Outcome Measures

There is no consensus definition of macrosomia, but being born large for gestational age (LGA) is often used [4]. The most common definition of LGA is a birth weight above the 90^th^ centile for a given gestational age. However, in Sweden, the definition of LGA is a birth weight of more than two standard deviations (approximately ≥ the 97^th^ centile) above national reference curves. In addition, an absolute birth weight above 4000 or 4500 g is sometimes used to define macrosomia [12]. This study’s primary outcome was a birth weight ≥ 90^th^ centile based on the national reference curve for gestational age and fetal sex [13]. Secondary outcomes were LGA defined by birth weight ≥ 97^th^ centile and birth weight ≥ 4000 and 4500 g, where the risks associated with fetal macrosomia increase sharply [14].

### Maternal Demographics and Characteristics

We collected data on maternal demographics and characteristics from the MBR. Based on previous studies [4, 5], we selected the following predictive variables for our models: maternal height, maternal weight, parity, country of birth, previous delivery of a newborn with macrosomia, smoking, history of chronic hypertension, and history of diabetes mellitus.

Maternal weight was measured at the first antenatal visit, and height was self-reported or measured. Smoking was self-reported at the first antenatal visit. Information on country of birth and previous delivery of a newborn with macrosomia was extracted from the MBR. Previous macrosomia was defined in the same way as for the outcome measure: birth weight ≥ 90^th^ centile for the primary outcome, or birth weight ≥ 97^th^ centile, 4000 and 4500 g for the secondary outcomes. History of chronic hypertension or type 1 or 2 diabetes mellitus was based on the corresponding International Classification of Diseases (ICD)−10 codes (O10, O11, I10-I15, O240, O241, E10, E11) assigned at discharge after delivery.

### Statistics

Descriptive data are presented as medians with interquartile range (IQR) for continuous variables and numbers and percentages for categorical variables.

Since the data were not normally distributed, we estimated the correlation between the continuous variables with Spearman´s rank correlation test. We compared waist circumference in women who gave birth to a newborn with macrosomia and women who did not with the Mann–Whitney U test.

We then determined the association of waist circumference and risk of a newborn with macrosomia using logistic regression analysis. Odds ratio (OR) and adjusted OR (AOR) with corresponding 95% confidence intervals (CIs) were estimated. We used already established predictors (as described above) and evaluated the influence of waist circumference and weight on the prediction of a newborn with macrosomia. The continuous variables waist circumference, weight and height were centered around the median. First, we included waist circumference and all predictors except weight. Second, we included weight and all predictors except waist circumference. In a third model, we included all predictors except waist circumference and weight. The logistic regression analyses were run on complete data sets. We imputed missing values for the predictors (no values were missing for waist circumference or weight) with chained equations approach using the predictive mean matching method [15].

To ensure that the link function of our logistic regression models was appropriate, we used the Pregibon Link Test. The test evaluates the adequacy of the link function (logit) by checking for misspecifications that could bias the models’ estimates.

To evaluate the value of waist circumference in the prediction model, we used C-statistical analysis and receiver operating characteristic (ROC) curves. The area under the curve (AUC), obtained following the logistic regression, was used to compare the models. To assess differences between the models we used the DeLong method for paired ROC curves [16]. Margin plots were used to visualize the relationship between waist circumference and weight and the predicted probability of macrosomia. The plots were derived from the predictive models, holding all other variables constant at their mean or reference values. Predicted probabilities were plotted against values corresponding to the 2.5^th^-97.5^th^ percentiles.

For the statistical analyses, we used the R language and environment for statistical computing (R Core Team, Vienna, Austria) version 4.2.3.

## Results

### Description of the Study Population

Of the 4423 included women, 489 (11.1%) had a newborn with macrosomia. The clinical characteristics of the study population are presented in Table 1.

**Table 1:** Demographic and clinical variables of the study population

Variable	Birth weight ≥ 90^th^ centile	
	Yes	No
	*N* = 489 (11.1)	*N* = 3934 (88.9)
Maternal age, years	31 (27, 35)	30 (27, 34)
Gestational age at waist measurement, weeks + days	9 + 5 (7 + 3, 10 + 5)	9 + 1 (7 + 2, 10 + 4)
Waist circumference, cm	86.0 (78.0, 95.0)	80.0 (74.0, 87.5)
Maternal weight, kg	73.0 (65.0, 85.0)	66.0 (59.0, 75.0)
Maternal height, cm	169 (164, 173)	166 (161, 170)
Country of birth		
- Sweden	425 (86.9)	2932 (74.5)
- Other Nordic countries	3 (0.6)	39 (1.0)
- Europe and North America	15 (3.1)	195 (5.0)
- All others	45 (9.2)	756 (19.2)
Parity		
- Nulliparous	131 (26.8)	1867 .5)
- Multiparous, no previous macrosomia	234 (47.9)	1773 .1)
- Multiparous, previous macrosomia	113 (23.1)	141 (3.6)
Smoking	19 (3.9)	171 (4.3)
Chronic hypertension	2 (0.4)	19 (3.9)
Type 1 or 2 diabetes mellitus	25 (5.1)	11 (0.3)

Data presented as the median and interquartile range for numeric variables or numbers and percentages for categorical variables

Waist circumference was measured at a median of 9 week + 5 days (IQR: 7 + 3, 10 + 5) gestational weeks for women with newborns with macrosomia and at a median of 9 + 1 (IQR: 7 + 2, 10 + 4) gestational weeks for those without. The women with newborns with macrosomia had a higher early pregnancy weight than women without. Further, women with a newborn with macrosomia were more often multiparous, had more often a history of previous birth of a newborn with macrosomia, and had more often type 1 or 2 diabetes mellitus.

### Correlation Between Waist Circumference and Weight

Waist circumference and weight in early pregnancy were positively correlated; rho = 0.80, *p* < 0.001. Maternal height was not strongly correlated to waist circumference or weight; rho < 0.4.

### Waist Circumference and Risk of Fetal Macrosomia

Women who gave birth to a newborn with macrosomia had a greater early pregnancy median waist circumference than women who did not (86.0 (IQR: 78.0, 95.0) vs. 80.0 (74.0, 87.5) cm, *p* < 0.001). The odds of having a newborn with macrosomia increased by 4% for every cm increase in waist circumference, crude OR 1.04 (95% CI 1.03, 1.05, *p* < 0.001).

In model 1, we adjusted for the following known predictive variables: maternal height, country of birth, parity, previous birth of a newborn weighing ≥ the 90^th^ centile, smoking, chronic hypertension, and type 1 or 2 diabetes mellitus. The adjusted odds of having a newborn with macrosomia increased by 3% for every cm increase in waist circumference, AOR 1.03 (95% CI 1.02, 1.04) (Table 2). In model 1, the baseline risk of macrosomia was 5.9%. For a 1 cm increase in waist circumference above the median, the risk increased to 6.1%. We also used waist circumference as a categorical variable. Compared with women with a waist circumference < 80 cm, those with a waist circumference between 80 and 88 cm had increased odds of having a newborn with macrosomia (AOR: 1.41,95% CI 1.09, 1.82,* p* < 0.01). For women with a waist circumference ≥ 88 cm, the odds were higher (AOR:1.98, 95% CI 1.56, 2.53, *p* < 0.001). In this model, the baseline risk of macrosomia was 4.9%. For women with a waist circumference of 80–88 cm, the risk increased to 6.7%, while for those with a waist circumference ≥ 88 cm, the risk was 9.2% (Supplementary Information).

**Table 2:** Logistic regression analysis for the prediction of having a newborn with macrosomia (birth weight ≥ 90^th^ centile)

Variable	Model 1, Waist circumference			Model 2, Weight			Model 3, No waist circumference or weight		
	Coefficient (Exponentiated Coefficient (95% CI))^2^	AOR (95% CI)	*p*-value	Coefficient (Exponentiated Coefficient (95% CI))^2^	AOR (95% CI)	*p*-value	Coefficient (Exponentiated Coefficient (95% CI))^2^	AOR (95% CI)	*p*-value
Intercept	−2.763 (0.063 (0.052, 0.077)		< .001	−2.772(0.063 (0.051, 0.075))		< .001	−2.717 (0.066 (0.054, 0.080)		< .001
Waist circumference^1^, cm		1.03 (1.02, 1.04)	< .001		-	-		-	-
Maternal weight^1^, kg		-	-		1.02 (1.01, 1.03)	< .001		-	-
Maternal height^1^, cm		1.05 (1.03, 1.07)	< .001		1.04 (1.02, 1.06)	< .001		1.06 (1.04, 1.07)	< .001
Country of birth: Other than Europe or North America		0.59 (0.42, 0.82)	< .01		0.60 (0.43, 0.84)	< .01		0.61 (0.43, 0.85)	< .01
Multiparous, no previous macrosomia		1.85 (1.48, 2.33)	< .001		1.96 (1.57, 2.47)	< .001		1.97 (1.57, 2.47)	< .001
Multiparous, previous macrosomia		8.77 (6.38, 12.1)	< .001		9.23 (6.73, 12.7)	< .001		10.5 (7.71, 14.4)	< .001
Smoking		0.68 (0.39, 1.12)	0.15		0.69 (0.40, 1.14)	0.17		0.81 (0.47, 1.323)	0.42
Chronic hypertension		0.43 (0.05, 1.91)	0.35		0.41 (0.05, 1.82)	0.32		0.51 (0.05, 2.30)	0.47
Type 1 or 2 diabetes mellitus		14.6 (6.81, 32.8)	< .001		15.6 (7.31, 35.0)	< .001		16.0 (7.51, 35.7)	< .001

Statistical analyses by logistic regression with having a newborn with macrosomia (birth weight ≥ 90^th^ centile) as the response variable. AOR is the adjusted odds ratio, i.e., the increased odds of having a newborn ≥ the 90^th^ centile for every unit increase in continuous variables. CI is confidence interval

^1^Waist circumference, weight and height are centered around the median

^2^The exponentiated intercept represents the odds of macrosomia when all continuous variables are at their median and categorical variables are at their reference levels

In model 2, we replaced waist circumference with weight to investigate if waist circumference could replace weight as a predictor for macrosomia. All other predictors from model 1 were kept. In model 2, the adjusted odds of having a newborn with macrosomia increased by 2% for every kg increase in maternal weight, AOR 1.02 (95% CI 1.01,1.03) (Table 2). In model 2, the baseline risk of macrosomia was 5.9%. For 1 kg increase in waist circumference above the median, the risk increased to 6.0%.

In the third model we included the predictors from model 1 and 2 but excluded both waist circumference and weight (Table 2).

We used the Pregibon Link Test to assess our models; the *p*-values associated with the squared term (η2) in our models for prediction of birth weight ≥ 90^th^ centile were: Model 1, 0.069; Model 2, 0.051; and Model 3, 0.50; indicating no strong evidence of link function misspecification.

### Waist Circumference in a Prediction Model for Fetal Macrosomia

**Fig. 2:**
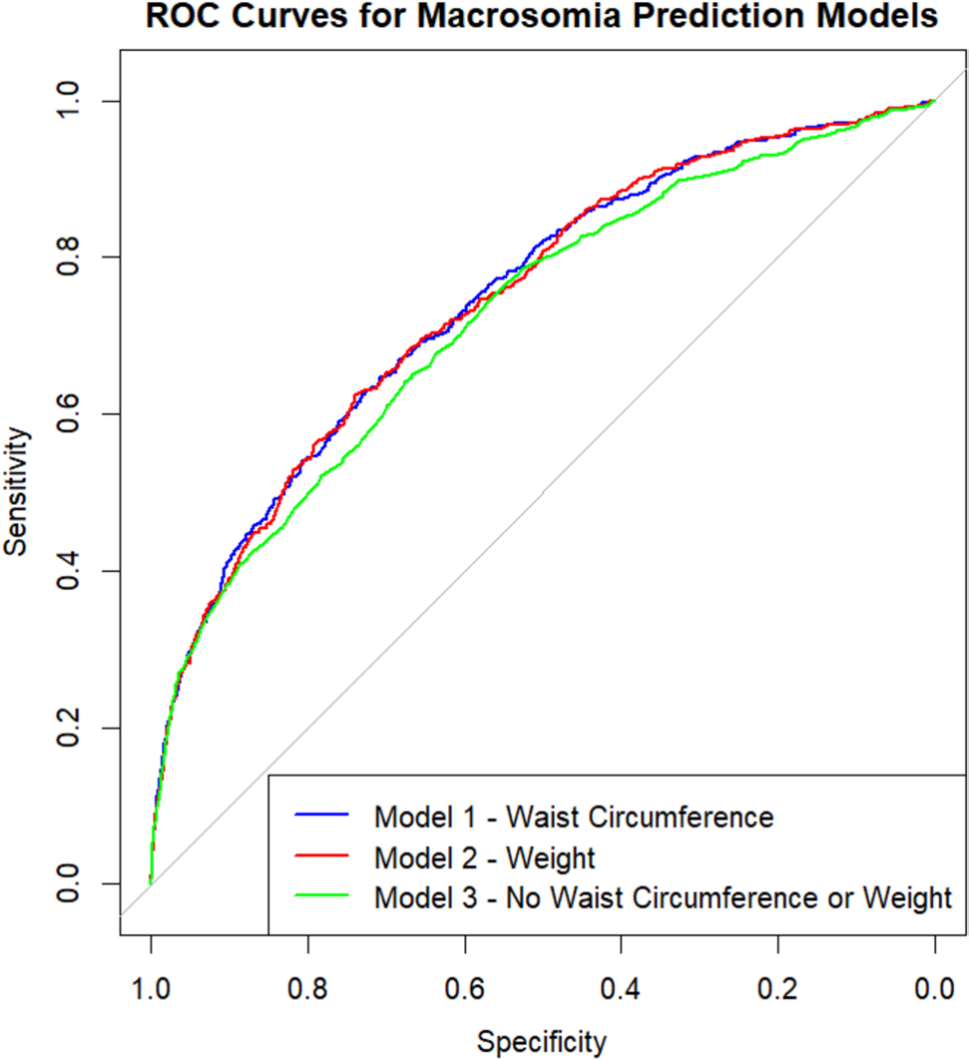
Receiver operating characteristic curves for the prediction models

**Fig. 3:**
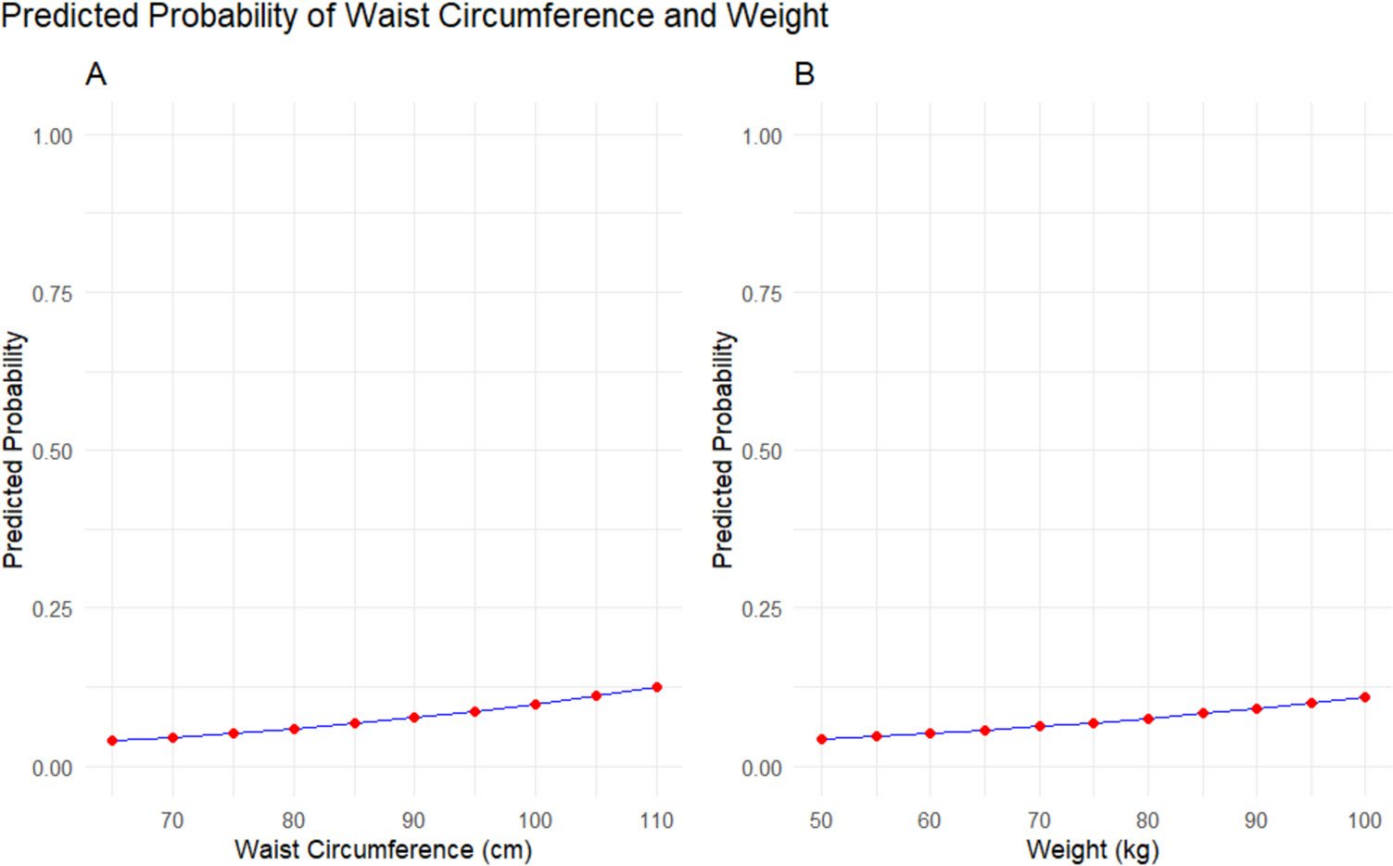
Predicted probabilities of macrosomia by **A** waist circumference and **B** maternal weight. Red points indicate model-predicted probabilities at each specified value, and the blue line shows the overall trend

We calculated and compared ROC curves for the odds of having a newborn with macrosomia to compare the three models’ predictive capacity (Fig. 2). In model 1, including waist circumference and all the other predictors except weight, the AUC was 0.75 (95% CI 0.72, 0.77). In model 2, including weight but not waist circumference, the AUC was, 0.74 (95% CI 0.72, 0.77). In model 3, without waist circumference and weight, the AUC was 0.72 (95% CI 0.70, 0.75). Accordingly, in model 1, the detection rates at false positive rates of 5 and 10% were 30.0 and 41.3%, respectively, and this was similar in the other two models. To test if waist circumference could be interchanged for weight, we compared the ROC curves for models 1 and 2, and there was no difference between the models (*p* = 0.68). However, both the model with waist circumference and the model with weight performed better than the model without waist circumference and weight (*p* < 0.001). To examine the relationship between the predictors and the likelihood of macrosomia, margin plots were generated. These plots display the predicted probability of macrosomia for waist circumference and weight holding all other variables constant (Fig. 3).

### Secondary Outcomes

For the secondary outcomes, LGA defined by birth weight ≥ 97^th^ centile, and birth weight ≥ 4 000 and 4500 g the results were similar to the primary outcome (Supplementary Information).

## Discussion

There is a need for a readily available method for fetal macrosomia prediction that could be used in low-resource settings. We found that early pregnancy maternal waist circumference can replace weight in a fetal macrosomia prediction model. Our model detected about 41% of women that gave birth to a newborn with macrosomia at a false-positive rate of 10%, similar to other studies of macrosomia prediction with maternal factors [4, 5].

We also compared the waist circumference model with a model without waist circumference and weight. Even though they had similar AUC values, there was a significant difference between the ROC curves, and the waist circumference model performed better. This difference suggests that including waist circumference positively affects classification at certain thresholds. However, it is important to note that both waist circumference and weight made relatively modest contributions to the model’s predictive capacity, raising questions about their clinical utility as predictors.

Our finding of a positive association between early pregnancy maternal waist circumference and fetal macrosomia is consistent with prior studies [9, 17–19]. However, to our knowledge, this is the first study evaluating waist circumference as an alternative to weight in an early pregnancy macrosomia prediction model with established predictors [4, 5].

Given the study’s design, we did not exclude outcomes such as preterm birth since these were not known at the time of the prediction. The macrosomia definition varies; therefore, we included several different definitions in the study. We chose our primary outcome (birth weight ≥ 90^th^ centile) as it is the most common definition of a large newborn. Nevertheless, since definitions of macrosomia varies, we also demonstrate that waist circumference could replace weight for predicting a newborn with a birth weight ≥ the 97^th^ centile, 4000 g, or 4500 g.

Obesity is a risk factor for fetal macrosomia and other adverse pregnancy outcomes. Obesity, defined by BMI or weight, is therefore used for risk stratification of pregnant women [12]. However, scales to measure weight are not available in all low-resource settings. Therefore, waist circumference could be an alternative measure of overweight and obesity in these settings. In addition, some women might have a negative attitude towards being weighted and could accept measuring waist circumference instead.

Moreover, BMI or weight does not account for the amount of muscle mass or fat distribution and does not directly estimate adiposity [20]. In fact, BMI only identifies 50% with adiposity-related risk in a non-pregnant population [21]. Waist circumference has therefore been suggested as a better measure of obesity-related risk in pregnancy than BMI [10]. However, it should be noted that waist circumference is a proxy for central adiposity and that other measures, such as ultrasound-measured fat mass are more exact [22]. The present study aimed to use a readily available measure, and in this context, waist circumference is a good choice. In our study, waist circumference was closely correlated with weight. As expected from this result, there was a similar predictive capacity of the model with waist circumference and the model with weight.

In contrast to previous studies [4, 5], smoking and having chronic hypertension, two of the predictors included in our model, did not affect the model significantly. In our study, 4% of included women smoked in early pregnancy, which is lower than in previous studies [4, 5] but comparable to Swedish pregnant women in general. In our study, 0.5% had chronic hypertension, comparable to what was previously reported from the MBR [23]. Similar to smoking, this prevalence is lower than in previous studies [4, 5]. The lower proportion of women who smoked or had chronic hypertension may explain why these presumed predictors did not affect our model significantly.

The major strength of our study is its large size and population-based design, which includes prospectively collected data. Further, using the MBR enabled us to include predictors such as the previous birth of a newborn with macrosomia. Moreover, waist circumference measurements were standardized. Waist circumference is influenced by the growing uterus later in pregnancy. It has been reported that the uterus starts affecting waist circumference at a uterine height of 27 cm, around 28 weeks of pregnancy [18, 24]. The median measurement time in this study was between gestational weeks 9 and 10 when the growing uterus has not reached the abdominal cavity.

One limitation of the study was the relatively few cases of smoking, chronic hypertension and diabetes. Another limitation is that the study is set in a high-resource setting and 87% of the women were born in Sweden. There might be differences in the associations between waist circumference and the other predictors and the risk of fetal macrosomia in other populations. Thus, our results may not be translatable to other settings. Moreover, in a low-resource setting, a woman might not attend a health center until later in the pregnancy, when the growing uterus makes waist circumference an inappropriate measure. Therefore, it would be valuable to make a similar study in a low-resource setting and include both waist circumference and other anthropometric measures such as mid-arm circumference.

In summary, we demonstrate that waist circumference can replace weight for macrosomia prediction. Our model could be applied in low-resource settings without access to fetal biometry, biochemical testing, and scales for measuring weight.

## Supplementary Information

Below is the link to the electronic supplementary material.

Supplementary file1 (DOCX 31.9 KB)

## Data Availability

The data that support the findings of this study are available from the corresponding author upon reasonable request.

## References

[1] Boulvain M, Irion O, Thornton JG. Induction of labour at or near term for suspected fetal macrosomia. Cochrane Database Syst. Rev. 2016;2016. 10.1002/14651858.CD000938.pub2 PMC7032677 27208913

[2] Beta J, et al. Maternal and neonatal complications of fetal macrosomia: systematic review and meta-analysis. Ultrasound Obstet Gynecol. 2019;54:308–18. 30938004 10.1002/uog.20279

[3] Wiebe HW, Boule NG, Chari R, Davenport MH. The effect of supervised prenatal exercise on fetal growth: a meta-analysis. Obstet Gynecol. 2015;125:1185–94. 25932847 10.1097/AOG.0000000000000801

[4] Poon LC, Karagiannis G, Stratieva V, Syngelaki A, Nicolaides KH. First-trimester prediction of macrosomia. Fetal Diagn Ther. 2011;29:139–47. 20798483 10.1159/000318565

[5] Frick AP, Syngelaki A, Zheng M, Poon LC, Nicolaides KH. Prediction of large-for-gestational-age neonates: screening by maternal factors and biomarkers in the three trimesters of pregnancy. Ultrasound Obstet Gynecol. 2016;47:332–9. 26446185 10.1002/uog.15780

[6] Ross R, et al. Waist circumference as a vital sign in clinical practice: a consensus statement from the IAS and ICCR working group on visceral obesity. Nat Rev Endocrinol. 2020;16:177–89. 32020062 10.1038/s41574-019-0310-7 PMC7027970

[7] Ebrahimi-Mameghani M, Mehrabi E, Kamalifard M, Yavarikia P. Correlation between body mass index and central adiposity with pregnancy complications in pregnant women. Health Promot Perspect. 2013;3:73–9. 24688955 10.5681/hpp.2013.009 PMC3963685

[8] Zhu Y, Hedderson MM, Quesenberry CP, Feng J, Ferrara A. Central obesity increases the risk of gestational diabetes partially through increasing insulin resistance. Obes Silver Spring. 2019;27:152–60. 10.1002/oby.22339 PMC6309219 30461219

[9] Li S, et al. Central adiposity and other anthropometric factors in relation to risk of macrosomia in an African American population. Obes Silver Spring. 2013;21:178–84. 10.1002/oby.20238 PMC3473111 23505184

[10] Nguyen G, et al. Association between maternal adiposity measures and infant health outcomes: A systematic review and meta-analysis. Obes Rev. 2022;23:e13491. 35801513 10.1111/obr.13491 PMC9539955

[11] World Health Organization. Waist circumference and waist-hip ratio : report of a WHO expert consultation. Geneva, 8-11 December 2008. 2011. https://apps.who.int/iris/handle/10665/44583

[12] Poston L, et al. Preconceptional and maternal obesity: epidemiology and health consequences. Lancet Diabetes Endocrinol. 2016;4:1025–36. 27743975 10.1016/S2213-8587(16)30217-0

[13] Marsal K, et al. Intrauterine growth curves based on ultrasonically estimated foetal weights. Acta Paediatr. 1996;85:843–8. 8819552 10.1111/j.1651-2227.1996.tb14164.x

[14] Boulet SL, Alexander GR, Salihu HM, Pass M. Macrosomic births in the united states: determinants, outcomes, and proposed grades of risk. Am J Obstet Gynecol. 2003;188:1372–8. 12748514 10.1067/mob.2003.302

[15] van Buuren S, Groothuis-Oudshoorn K. Mice: Multivariate imputation by chained equations in R. J Stat Softw. 2011;45:1–67.

[16] DeLong ER, DeLong DM, Clarke-Pearson DL. Comparing the areas under two or more correlated receiver operating characteristic curves: A nonparametric approach. Biometrics. 1988;44:837–45. 3203132

[17] Mehrabi E, Kamalifard M, Yavarikia P, Ebrahimi Mameghani M. The relation between early pregnancy anthropometric indices among primiparous women and macrosomia. J Caring Sci. 2012;1:153–8. 25276690 10.5681/jcs.2012.022 PMC4161076

[18] Wendland EMDR, Duncan BB, Mengue SS, Nucci LB, Schmidt MI. Waist circumference in the prediction of obesity-related adverse pregnancy outcomes. Cad Saude Publica. 2007;23:391–8. 17221088 10.1590/s0102-311x2007000200015

[19] Gao X, et al. The mutual effect of pre-pregnancy body mass index, waist circumference and gestational weight gain on obesity-related adverse pregnancy outcomes: A birth cohort study. PLoS One. 2017;12:e0177418. 28575041 10.1371/journal.pone.0177418 PMC5456032

[20] National Clinical Guideline Centre (UK). Obesity: Identification, assessment and management of overweight and obesity in children, young people and adults: partial update of CG43. National Institute for Health and Care Excellence (NICE). 2014. http://www.ncbi.nlm.nih.gov/books/NBK264165/. 25535639

[21] Okorodudu DO, et al. Diagnostic performance of body mass index to identify obesity as defined by body adiposity: a systematic review and meta-analysis. Int J Obes. 2010;34:791–9. 10.1038/ijo.2010.5 20125098

[22] Lindberger E, Ahlsson F, Junus K, Wikström A-K, Sundström Poromaa I. Combined maternal central adiposity measures in relation to infant birth size. Sci Rep. 2024;14:725. 38184682 10.1038/s41598-024-51274-6 PMC10771412

[23] Sohlberg S, Stephansson O, Cnattingius S, Wikström A-K. Maternal body mass index, height, and risks of preeclampsia. Am J Hypertens. 2012;25:120–5. 21976280 10.1038/ajh.2011.175

[24] Branchtein L, et al. Waist circumference and waist-to-hip ratio are related to gestational glucose tolerance. Diabetes Care. 1997;20:509–11. 9096970 10.2337/diacare.20.4.509

