# Supplementary material for: Early Pregnancy Waist Circumference for Prediction of Fetal Macrosomia
Source: Reprod Sci. 2025 Mar 10;32(4):1072–9. doi: 10.1007/s43032-025-01833-7 (PMC11978530; doi:10.1007/s43032-025-01833-7)
Supplement: Supplementary file 1 — (DOCX 31.9 KB) [file 43032_2025_1833_MOESM1_ESM.docx]

**Supplementary Information**

**Table S1.** Logistic regression analysis for the prediction of having a newborn with macrosomia (birth weight ≥ 90^th^ centile) using waist circumference as a categorical variable

|  |  | | |
| --- | --- | --- | --- |
| Variable | Coefficient  (Exponentiated Coefficient (95% CI))^2^ | AOR (95% CI) | p-value |
| Intercept | -2.970  (0.051 (0.041,0.064)) |  | <.001 |
| Waist circumference, 80 - 88 cm |  | 1.41 (1.09, 1.82) | <.01 |
| Waist circumference, ≥ 88 cm |  | 1.98 (1.56, 2.53) | <.001 |
| Maternal height^1^, cm |  | 1.05 (1.03, 1.07) | <.001 |
| Country of birth: Other than Europe or North America |  | 0.59 (0.42, 0.82) | <.01 |
| Multiparous, no previous macrosomia |  | 1.85 (1.48, 2.33) | <.001 |
| Multiparous, previous macrosomia |  | 9.01 (6.56, 12.4) | <.001 |
| Smoking |  | 0.73 (0.42, 1.19) | 0.22 |
| Chronic hypertension |  | 0.45 (0.05, 2.06) | 0.40 |
| Type 1 or 2 diabetes mellitus |  | 15.7 (7.34, 35.15) | <.001 |
| Statistical analyses by logistic regression with having a newborn with macrosomia (birth weight ≥ 90^th^ centile) as the response variable. AOR is the adjusted odds ratio, i.e., the increased odds of having a newborn ≥ the 90^th^ centile for every unit increase in continues variables. CI is confidence interval.  ^1^Maternal height is centered around the median.  ^2^The exponentiated intercept represents the odds of macrosomia when maternal height is at median and categorical variables are at their reference levels. | | | |

**Table S2**. Logistic regression analysis for the prediction of having a newborn with birth weight ≥ 97^th^ centile

|  | Model 1, Waist Circumference | | | Model 2, Weight | | | Model 3, No Waist Circumference or Weight | | |
| --- | --- | --- | --- | --- | --- | --- | --- | --- | --- |
| Variable | Coefficient  (Exponentiated Coefficient (95% CI))^2^ | AOR (95% CI) | p-value | Coefficient  (Exponentiated Coefficient)^2^ | AOR (95% CI) | p-value | Coefficient  (Exponentiated Coefficient)^2^ | AOR (95% CI) | p-value |
| Intercept | -3.925  (0.020 (0.014, 0.027)) |  | <.001 | -3.971  (0.019 (0.013, 0.026)) |  | <.001 | -3.851  (0.021 (0.015, 0.029)) |  | <.001 |
| Waist circumference^1^, cm |  | 1.03 (1.02, 1.05) | <.001 |  | - | - |  | - | - |
| Maternal weight^1^, kg |  | - | - |  | 1.03 (1.02, 1.04) | <.001 |  | - | - |
| Maternal height^1^, cm |  | 1.04 (1.02, 1.07) | <.01 |  | 1.03 (1.02, 1.04) | <.05 |  | 1.05 (1.02, 1.08) | <.001 |
| Country of birth: Other than Europe or North America |  | 0.35 (0.16, 0.65) | <.01 |  | 0.36 (0.17, 0.68) | <.01 |  | 0.37 (0.18, 0.70) | <.01 |
| Multiparous, no previous newborn with birth weight ≥ 97th centile |  | 1.94 (1.35, 2.83) | <.001 |  | 2.08 (1.45, 3.03) | <.001 |  | 2.13 (1.49, 3.10) | <.001 |
| Multiparous, previous newborn with birth weight ≥ 97th centile |  | 20.5 (11.8, 35.6) | <.001 |  | 22.7 (13.1, 39.2) | <.001 |  | 26.4 (15.4, 45.3) | <.001 |
| Smoking |  | 0.45 (0.16, 1.04) | 0.09 |  | 0.45 (0.17, 1.02) | 0.08 |  | 0.59 (0.22, 1.31) | 0.24 |
| Chronic hypertension |  | 0.68 (0.04, 3.83) | 0.73 |  | 0.62 (0.03, 3.44) | 0.66 |  | 0.82 (0.04, 4.70) | 0.86 |
| Type 1 or 2 diabetes mellitus |  | 12.8 (5.70, 28.1) | <.001 |  | 14.2 (6.30, 31.1) | <.001 |  | 14.5 (6.49, 31.4) | <.001 |
| Statistical analyses by logistic regression with having a newborn with birth weight ≥ 97t^h^ centile as the response variable. AOR is the adjusted odds ratio, i.e., the increased odds of having a newborn ≥ the 97^th^ centile for every unit increase in continues variables. CI is confidence interval.  ^1^Waist circumference, weight and height are centered around the median.  ^2^The exponentiated intercept represents the odds of macrosomia when all continuous variables are at their median and categorical variables are at their reference levels. | | | | | | | | | |

**Table S3**. Logistic regression analysis for the prediction of having a newborn with birth weight ≥ 4000 g

|  | Model 1, Waist Circumference | | | Model 2, Weight | | | Model 3, No Waist Circumference or Weight | | |
| --- | --- | --- | --- | --- | --- | --- | --- | --- | --- |
| Variable | Coefficient  (Exponentiated Coefficient)^2^ | AOR (95% CI) | p-value | Coefficient  (Exponentiated Coefficient)^2^ | AOR (95% CI) | p-value | Coefficient  (Exponentiated Coefficient)^2^ | AOR (95% CI) | p-value |
| Intercept | -1.886  (0.152 (0.132, 0.174)) |  | <.001 | -1.902  (0.149 (0.130, 0.171)) |  | <.001 | -1.869  (0.154 (0.134, 0.176)) |  | <.001 |
| Waist circumference^1^, cm |  | 1.02 (1.01, 1.03) | <.001 |  | - | - |  | - | - |
| Maternal weight^1^, kg |  | - | - |  | 1.02 (1.01, 1.02) | <.001 |  | - | - |
| Maternal height^1^, cm |  | 1.05 (1.03, 1.06) | <.001 |  | 1.04 (1.02, 1.05) | <.001 |  | 1.05 (1.04, 1.06) | <.001 |
| Country of birth: Other than Europe or North America |  | 0.77 (0.60, 0.99) | <.05 |  | 0.78 (0.61, 1.00) | 0.05 |  | 0.79 (0.61, 1.01) | 0.06 |
| Multiparous, no previous newborn with birth weight ≥ 4000 g |  | 1.14 (0.95, 1.37) | 0.15 |  | 1.18 (0.99, 1.42) | 0.07 |  | 1.19 (0.99, 1.42) | 0.06 |
| Multiparous, previous newborn with birth weight ≥ 4000 g |  | 5.83 (4.67, 7.30) | <.001 |  | 5.98 (4.79, 7.48) | <.001 |  | 6.42 (5.15, 8.01) | <.001 |
| Smoking |  | 0.69 (0.44, 1.04) | 0.09 |  | 0.68 (0.44, 1.03) | 0.08 |  | 0.77 (0.49, 1.15) | 0.22 |
| Chronic hypertension |  | 0.42 (0.07, 1.50) | 0.25 |  | 0.40 (0.06, 1.42) | 0.22 |  | 0.48 (0.08, 1.71) | 0.34 |
| Type 1 or 2 diabetes mellitus |  | 3.06 (1.49, 6.21) | <.01 |  | 3.23 (1.57, 6.55) | <.01 |  | 3.37 (1.64, 6.83) | <.001 |
| Statistical analyses by logistic regression with having a newborn with birth weight ≥ 4000 g as the response variable. AOR is the adjusted odds ratio, i.e., the increased odds of having a newborn ≥ 4000 g for every unit increase in continues variables. CI is confidence interval.  ^1^Waist circumference, weight and height are centered around the median.  ^2^The exponentiated intercept represents the odds of macrosomia when all continuous variables are at their median and categorical variables are at their reference levels. | | | | | | | | | |

**Table S4**. Logistic regression analysis for the prediction of having a newborn with birth weight ≥ 4500 g

|  | Model 1, Waist Circumference | | | Model 2, Weight | | | Model 3, No Waist Circumference or Weight | | |
| --- | --- | --- | --- | --- | --- | --- | --- | --- | --- |
| Variable | Coefficient  (Exponentiated Coefficient)^2^ | AOR (95% CI) | p-value | Coefficient  (Exponentiated Coefficient)^2^ | AOR (95% CI) | p-value | Coefficient  (Exponentiated Coefficient)^2^ | AOR (95% CI) | p-value |
| Intercept | -3.881  (0.021 (0.015, 0.028)) |  | <.001 | -3.903  (0.021 (0.015, 0.027)) |  | <.001 | -3.830  (0.022 (0.016, 0.029)) |  | <.001 |
| Waist circumference^1^, cm |  | 1.03 (1.01, 1.04) | <.001 |  | - | - |  | - | - |
| Maternal weight^1^, kg |  | - | - |  | 1.02 (1.01, 1.03) | <.001 |  | - | - |
| Maternal height^1^, cm |  | 1.08 (1.05, 1.11) | <.001 |  | 1.07 (1.04, 1.10) | <.001 |  | 1.09 (1.06, 1.12) | <.001 |
| Country of birth: Other than Europe or North America |  | 0.76 (0.41, 1.29) | 0.33 |  | 0.77 (0.42, 1.32) | 0.36 |  | 0.77 (0.42, 1.32) | 0.37 |
| Multiparous, no previous newborn with birth weight ≥ 4500 g |  | 1.45 (1.01, 2.09) | <.05 |  | 1.53 (1.07, 2.21) | <.05 |  | 1.56 (1.10, 2.25) | <.05 |
| Multiparous, previous newborn with birth weight ≥ 4500 g |  | 14.7 (8.6, 25.0) | <.001 |  | 15.3 (8.95, 25.9) | <.001 |  | 17.3 (10.2, 29.2) | <.001 |
| Smoking |  | 0.52 (0.17, 1.22) | 0.18 |  | 0.52 (0.18, 1.22) | 0.18 |  | 0.64 (0.22, 1.47) | 0.35 |
| Type 1 or 2 diabetes mellitus |  | 2.99 (0.95, 7.74) | <.05 |  | 3.32 (1.06, 8.50) | <.05 |  | 3.45 (1.10, 8.87) | <.05 |
| Statistical analyses by logistic regression with having a newborn with birth weight ≥ 4500 g as the response variable. AOR is the adjusted odds ratio, i.e., the increased odds of having a newborn ≥ 4500 g for every unit increase in continues variables. CI is confidence interval.  ^1^Waist circumference, weight and height are centered around the median.  ^2^The exponentiated intercept represents the odds of macrosomia when all continuous variables are at their median and categorical variables are at their reference levels. Chronic hypertension not included in models due to few cases | | | | | | | | | |
